# *Leishmania* Protein Kinases: Important Regulators of the Parasite Life Cycle and Molecular Targets for Treating Leishmaniasis

**DOI:** 10.3390/microorganisms9040691

**Published:** 2021-03-27

**Authors:** Antonia Efstathiou, Despina Smirlis

**Affiliations:** Microbiology Department, Hellenic Pasteur Institute, Avenue Vas. Sofias 127, 11521 Athina, Greece; penny@pasteur.gr

**Keywords:** *Leishmania*, leishmaniasis, protein kinases, cell cycle, drug targets, MAPKs, CDKs, GSK-3, DYRKs, trypanosomatids

## Abstract

*Leishmania* is a protozoan parasite of the trypanosomatid family, causing a wide range of diseases with different clinical manifestations including cutaneous, mucocutaneous and visceral leishmaniasis. According to WHO, one billion people are at risk of *Leishmania* infection as they live in endemic areas while there are 12 million infected people worldwide. Annually, 0.9–1.6 million new infections are reported and 20–50 thousand deaths occur due to *Leishmania* infection. As current chemotherapy for treating leishmaniasis exhibits numerous drawbacks and due to the lack of effective human vaccine, there is an urgent need to develop new antileishmanial therapy treatment. To this end, eukaryotic protein kinases can be ideal target candidates for rational drug design against leishmaniasis. Eukaryotic protein kinases mediate signal transduction through protein phosphorylation and their inhibition is anticipated to be disease modifying as they regulate all essential processes for *Leishmania* viability and completion of the parasitic life cycle including cell-cycle progression, differentiation and virulence. This review highlights existing knowledge concerning the exploitation of *Leishmania* protein kinases as molecular targets to treat leishmaniasis and the current knowledge of their role in the biology of *Leishmania* spp. and in the regulation of signalling events that promote parasite survival in the insect vector or the mammalian host.

## 1. Introduction

### Leishmaniases and Current Chemotherapy

*Leishmania*, a kinetoplastid protozoan parasite which belongs to the order of trypanosomatids together with *Trypanosoma brucei* and *Trypanosoma cruzi*, is the causative agent of leishmaniases, an umbrella term of diseases which includes cutaneous, mucocutaneous and visceral leishmaniasis [1].

*Leishmania* parasites are transmitted to the mammalian host after a bite of an infected female sandfly. *Leishmania spp* have a digenetic life cycle, involving a motile, extracellular promastigote stage that parasitizes the alimentary tract of a sandfly vector and an immotile amastigote stage that survives and replicates in the phagolysosomes of mononuclear phagocytes [2,3]. Apart from humans, primary hosts of *Leishmania* are vertebrates, including wild and domestic animals such as rodents and canines [4].

A main factor that contributes to the broad spectrum of disease manifestation is the diversity of *Leishmania* species [4,5]. More than twenty *Leishmania* species inflict disease with distinct clinical manifestations and are responsible for 10–12 million infected people worldwide. Annually, 0.9–1.6 million new infections and 20–50 thousand deaths are reported due to *Leishmania* infection [5]. *Leishmania* (*L.) amazonensis*, *L. major*, *L. mexicana* and *L. tropica* are associated with cutaneous leishmaniasis with reported cases in 92 countries [1]. Cutaneous leishmaniasis (CL), the most common form of the disease, causes self-curing lesions. Depending on the parasite species, the host immune response and the vector, some cases of cutaneous leishmaniasis lead to more severe manifestations of the disease such as mucocutaneous or diffuse cutaneous leishmaniasis [5,6]. *L. braziliensis* and *L. guyanensis* cause mucocutaneous leishmaniasis, mostly to immunocompromised individuals, leading to disability outcomes due to disfigurement lesions, primarily to the nose and lips, and to potentially life threating symptoms such as aspiration pneumonia [7,8]. The most severe form of the disease, visceral leishmaniasis is associated with *L. donovani* (Asia, Africa) and *L. infantum* (Mediterranean, Middle East, Central and South America) and is responsible for 30,000 new cases annually in 83 countries worldwide [1,5]. Visceral leishmaniasis (VL) is usually fatal without treatment after symptoms of fever, weight loss, anemia, splenomegaly and hepatomegaly. Another important disease manifestation is post-kala-azar dermal leishmaniasis, a post-treatment manifestation affecting 5–10% of VL cases [1].

Leishmaniases are strongly associated with geographical and socioeconomic factors, affecting the poorest of the poor, with 1 billion people being at risk of infection, mostly in endemic rural regions, thus classified as neglected tropical diseases (NTDs) [1]. Drug discovery for NTDs is limited as drugs used for diseases in the poorest regions of the world will provide little or no profits to pharmaceutical companies. Consequently, there are only a few drugs available for leishmaniasis treatment and have serious limitations like toxicity, effectiveness or inconvenience in administration. The basic chemotherapy used against leishmaniasis, pentavalent antimonial compounds display toxicity, while antimony resistance is progressively increasing in the endemic areas [9]. Amphotericin B deoxycholate emerged in 1950s as an alternative treatment for VL, however it has the drawback of being administered by slow intravenous infusion and it is highly toxic [10]. To overcome the antimony resistance and the high toxicity, ambisome, a liposomal formulation of amphotericin B, is used as a second line treatment [4,9]. Nevertheless, ambisome is expensive and patients in endemic areas cannot afford the treatment. Paromomycin, an aminoglycoside antibiotic, is used for the treatment of CL and VL cases, but the cure rates seem to have heterogenicity depending on the geographic region [11]. In order to overcome the drawback of lower cure rates of paromomycin and the toxicity of pentavalent antimonials, a combinatorial therapy is recommended by WHO in certain populations [10,12]. Moreover, treatment with pentamidine, which displays numerous toxic effects such as cardiotoxicity, was “a forgotten” leishmanial therapy which re-emerged only after the increasing antimonial resistance. However, pentamidine treatment also displays unsatisfactory cure rates in VL cases [13]. Thus, scientists often focus their efforts in repurposing drugs that are used for the treatment of other diseases, in order to treat NTDs, as this approach can limit the cost. Miltefosine, an orally administrated anticancer drug, is a relatively recent example of a successful repurposing treatment for leishmaniasis [14,15], but even this new drug has the potential for teratogenesis and drug failure due to increasing drug resistance [16].

As current chemotherapy displays numerous drawbacks and there is no human vaccine against leishmaniasis, the need to discover new effective chemotherapy is urgent. To this end, combinatorial therapy studies against cutaneous and visceral leishmaniasis are increasingly emerging [17,18,19] while repurposing drugs such as artesunate, an effective drug for the treatment of malaria, is a rational approach to fight against the disease [20,21,22,23]. At the same time, many research groups show an interest in natural products for uncovering new chemotherapies, in order to minimize potential side effects. For instance, artesunate, the most stable derivative of the sequiterpene lactone artemisin which is derived from a Chinese plant, seems to possess antileishmanial activity and to prevent pain and neuroinflammation induced by *L. amazonensis* in BALB/c mice [20]. Amongst others, indirubins, a family of naturally occurring bis-indole compounds which were used for centuries in traditional Chinese medicine and are found in indigo-bearing plants (*Isatis* spp., *Polygonum* spp.) and in marine organisms (Murex shellfish family, *Hexaplex trunculus*) [24], are potent anti-trypanosomatid agents [25,26,27,28,29], while total phenolic fraction of extra virgin olive oil, a key product in the Mediterranean diet, exhibited antileishmanial activity and *in vivo* induction of T cell-mediated responses in experimental cutaneous leishmaniasis [30]. In addition, more and more research studies are oriented towards targeted drug discovery, after validating targets that are essential for parasite viability and/or infectivity. The completion of the genome sequencing of *Leishmania* strengthens these efforts and provides insights into important pathways for parasite viability and/or infectivity and drug target prediction and validation [31,32]. Amongst these, an important family of proteins that can serve as molecular targets to treat leishmaniasis are the *Leishmania* eukaryotic protein kinases (ePKs) [33].

## 2. Eukaryotic Protein Kinases (ePKs) as Potential Drug Targets

In humans, ePKs are an important source of drug targets against a variety of diseases and rank only second after G-protein coupled receptors. In mammalian cells, ePKs play an essential role in modulating almost all cellular processes. Currently, more than 28 kinase inhibitors are approved by FDA for the treatment of human diseases [34]. In *Leishmania*, as in all eukaryotes, ePKs regulate essential functions in the cell, including cell cycle progression, differentiation and virulence, and thus inhibition is anticipated to be disease modifying [33]. Thus, as *Leishmania spp* do not encode for G-protein coupled receptors, ePKs are placed in the centre of attention for the validation of novel drug targets and drug discovery efforts. 

The leishmanial kinome consists of 175–195 eukaryotic protein kinases (ePKs) depending on the *Leishmania* species and represents roughly 2% of the encoded proteins [35,36,37]. Catalytic domain similarity according to Manning et al., has classified *Leishmania* ePKs in several groups, namely CMGC, AGC, CAMK, CK1, STE and “other” (several kinase families that do not fit within any of the other main kinase groups) [38]. Notably, the *Leishmania* genome does not encode for ePKs that belong to tyrosine kinases, tyrosine kinase-like (TKL) and receptor guanylate cyclase (RGC) groups while dual specificity kinases are present [35,36]. 

In recent years, great advances have been made by genetic or chemical approaches to validate members’ ePKs as potential drug targets and to study their role in the parasite life cycle [37]. In general, at the centre of attention are members of the CMGC group, namely the Cyclin-dependant kinases (CDKs), glycogen synthase kinase 3 (GSK-3), dual-specificity tyrosine-regulated kinases (DYRKs), mitogen-activated protein kinases (MAPKs). In addition, Casein kinases (CKs) and Aurora kinases have also attracted attention for drug discovery efforts. Besides studies on specific ePKs, recently a kinome-wide gene deletion and localization study in *L. mexicana* was carried out to establish the importance of leishmanial ePKs in *Leishmania* survival, differentiation and infection [37]. In this study, the library of protein kinase gene deletion mutants was pooled and requirements through life cycle stages, using bar-seq technology, were investigated [37]. Overall, 21% of all kinases and 25% of CMCGs were refractory to deletion, suggesting that many of these ePKs could be essential for survival [37]. Amongst these ePKs, there were several kinases which have already been shown to be essential for viability in previous studies. This study also identified 15 ePKs that are required for colonization in the sand-fly and 29 ePKs that are required for successful differentiation from metacyclic promastigote to amastigote form or for macrophage infection and a further 15 for infection *in vivo*. Thus, ePK genes that are required for amastigote proliferation and/or infectivity, as well as those which are refractory to deletion [37] could be further investigated as potential drug targets.

## 3. Protein Kinase Domains and Kinase Inhibition

**Figure 1:**
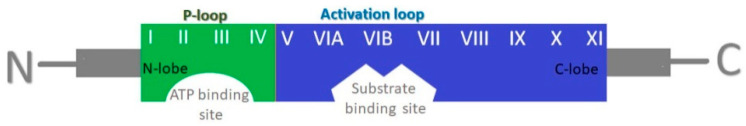
Protein kinase general structure. The N-terminal lobe (green) and the larger C-terminal lobe (blue) of the kinase are displayed in the figure. N-lobe includes the ATP binding pocket and the P-loop while activation loop and the Substrate Binding site are present in the C-lobe. The 12 conserved kinase subdomains (I, II, III, IV, V, VIA, VIB, VII, VIII, IX, X and XI) are also displayed.

ePKs catalyze the transfer of the gamma-phosphate from ATP to the hydroxyl group of a Ser, Thr, or Tyr of a substrate protein. The catalytic activity of these enzymes is associated with a relatively well conserved catalytic core. Hanks and Hunter showed that the kinase domain contains 12 conserved subdomains (I, II, III, IV, V, VIA, VIB, VII, VIII, IX, X and XI) that fold into a common catalytic core structure, as revealed by the 3-dimensional structures of several protein-serine kinases [39]. All ePKs in their active form adopt strikingly similar structures. Overall, the 3D structure of ePKs is comprised of two lobes (N-terminal and C-terminal) connected with a flexible hinge, while the active site is located in a cleft between the lobes [40,41,42,43,44]. More specifically, the N-terminal (N-) lobe is smaller and contains a β-sheet structure and a helix, named helix C, while the larger C-terminal (C-) lobe is mainly α-helical with conserved helices E and F in the core. Moreover, protein kinases possess two important motifs, the glycine-rich phosphate-binding (P-) loop involved in ATP binding, and the activation (A-) loop, where the peptide substrate usually binds (Figure 1) [45].

Helix C plays a crucial role in the modulation of the kinase activity as it is coupled to both the ATP binding site and the activation loop. Helix C can rotate in response to regulators and subsequently reconstitutes the ATP binding site promoting the active form of the kinase when there is simultaneously a phosphorylation of the activation loop [45]. The activation loop is a complex domain in the kinase structure, and when reconstructed in its active form upon phosphorylation, it allows the substrate binding. The activation loop is therefore a part of the substrate binding site and is flexible in order to accommodate the ATP binding site [45]. Finally, a gatekeeper residue partially or fully blocks a hydrophobic region in the ATP binding pocket and is considered as a selectivity determinant of most ATP competitive kinase inhibitors [46].

Blocking the conserved ATP binding site is the most common mechanism to inhibit the kinase, however additional structures can be exploited for the inhibition of kinase activity. For instance, the fact that the substrate binding site can be blocked via intrasteric interactions or modulated by the conformation of the activation loop, can be useful for designing molecules interacting with those domains to block the activation of the kinase. Moreover, the flanking segments of the kinase can be responsible for autoinhibition by blocking the active site or by promoting conformational change in the kinase. Thus, flanking segments could also be targeted for modifying the kinase in its inactive structure [45].

## 4. Cyclin-Dependent Kinases (CDKs)

Cyclin-dependent kinases (CDKs) belong to the group CMGC kinases, and like their name reveals, possess a cyclin-binding region, for the binding of a cyclin partner which is required for kinase activation. CDKs are found in all eukaryotes and are key regulators of cell cycle progression and other processes, including transcription, mRNA processing and differentiation of nerve cells [47]. Animal cells contain at least 20 CDKs, four of which, namely CDK1, CDK2, CDK3 and CDK4, are directly involved in cell cycle regulation [48]. CDK1 was the first CDK identified and plays important role in mitosis, while it can drive S phase in the absence of CDK2. In higher eukaryotes, CDK2 and CDK3 are essential for the G1 phase of the cell cycle [49,50,51,52]. Kinases that belong to this group have been extensively studied and targeted for the treatment of various diseases including various types of human cancers [53]. In the trypanosomatid kinome, CDKs are over-represented. The *Leishmania* genome encodes for 11 CDKs referred as CDC2-related kinases (CRKs) and 11 activating cyclins (CYCA, CYC2-CYC11) [35].

**Figure 2:**
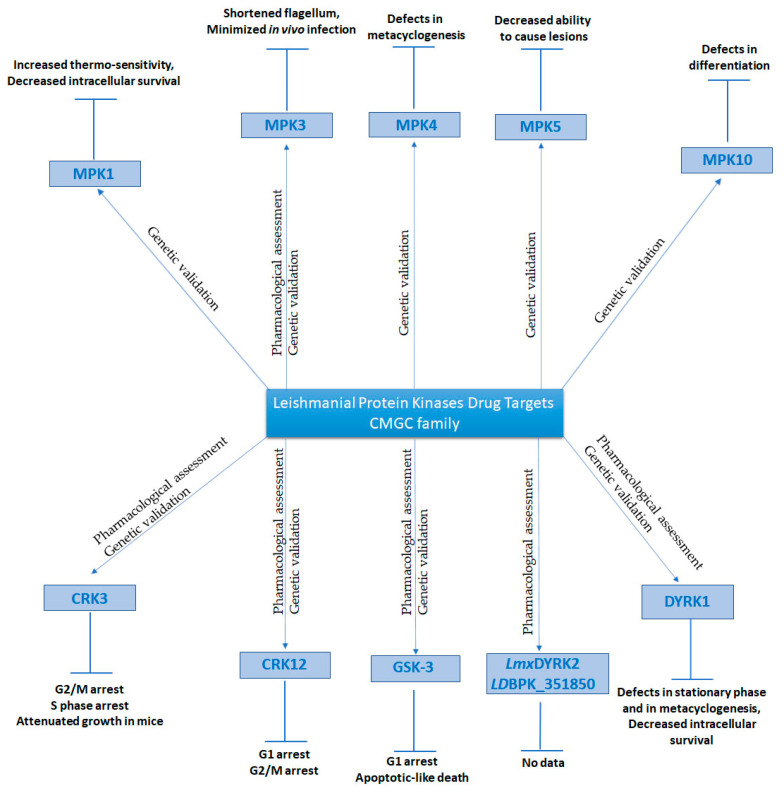
Leishmanial Protein kinases from CMGC family that could serve as drug targets. The ePKs displayed have been genetically and/or pharmacologically validated. The effects on their biological role and/or on their virulence upon pharmacological and/or genetic inhibition are also displayed in the diagram.

The most studied CRK in *Leishmania* is the leishmanial homologue of the mammalian CDK1, namely CRK3 (*Lmx*M.36.0550 in *L. mexicana*). Gene disruption studies carried out in *L. mexicana* demonstrated that both CRK3 alleles can be replaced, although ploidy changes occurred to allow retention of the wild-type copy of CRK3, suggesting but not confirming that CRK3 is an essential enzyme [54]. The essentiality of this kinase was confirmed later with the use of a rapamycin-inducible gene deletion system, based on a dimerized Cre recombinase (diCre) [55]. Induction of diCre activity in promastigotes with rapamycin resulted in G2/M growth arrest, confirming the essentiality of this kinase in mitotic transitions [56]. Inducible deletion of CRK3 in stationary phase promastigotes resulted in attenuated growth in mice, thus genetically validating CRK3 as a drug target to treat leishmaniasis (Figure 2) [56].

Studies have highlighted that both the kinase and the cyclin binding partner of CRK3 are essential for its activity and thus are targeted as a complex by potential inhibitors [57]. In *Leishmania*, CRK3 is known to complex with CYC1, CYCA and CYC6 cyclins [33,58,59,60]. Μost inhibitor screens, however, are focused on the CRK3-CYC6 complex as it is thought to have homologous function to the cyclin-dependent kinase 1–cyclin B complex (CDK1–CYCB) in humans, which is essential for the proliferation and coordination of the eukaryotic cell cycle [61,62,63]. While CRK3 is required for the *Leishmania* G2/M transition possibly via CRK3-CYC6 complex formation, the CRK3-CYC1 is an S phase complex highlighting additional roles of CRK3 in the S phase of the cell cycle (Figure 2). Although the importance and the role of CRK3-CYC1 substrates is yet to be determined, their identification could serve as a scaffold for generating inhibitors to screen the CRK3-CYC1 complex and to further investigate the role of CRK3 in *Leishmania* [64]. 

Up to date, there are a number of reports on inhibitors of CRK3 and novel antileishmanial compounds [25,26,27,62,63] (Table 1). Amongst the most potent inhibitors are a family of bis indole compounds, the indirubins, known inhibitors of CDKs and GSK3s including 5-substituted and 6-substituted indirubins, inhibiting CRK3 at nM concentrations [25,26]. In addition, research studies have identified 2,6,9-trisubstituted purines [61], triazolopyridine inhibitors [62], azapurine and thiazole compounds [63], disubstituted purines and pyrimidines [65], paullones and staurosporine derivatives [25] as CRK3 inhibitors in *Leishmania* (Table 1). Among these CRK3 inhibitors, disubstituted and trisubstituted purines, disubstituted pyrimidines and indirubin analogues displayed antileishmanial activity *in vitro* while thiazole compounds showed only moderate activity against *Leishmania* parasites [25,26,61,63,65]. 

In addition to CRK3, CRK12 (*Ld*BPK_090270) was chemically validated as a drug target for VL, with the use of a chemical series based on a pyrazolopyrimidine scaffold (Table 1), while CYC9 emerged as the definite partner of CRK12 [66]. A gene deletion mutant could not be generated without ectopic expression of CRK12, implying that CRK12 could be an essential *Leishmania* enzyme (Figure 2). Moreover, the leading compound of the series could block the cell cycle in G1 and G2/M phases, suggesting that CRK12 is important for the cell cycle progression through these phases [66]. 

In addition to CRK12, attempts to generate *Lmx*CRK1 (*Lmx*M.21.1080) null mutants in *L. mexicana* promastigotes failed unless compensated with a *T. brucei* CRK1 homologue, thus strongly suggesting that this gene was essential for promastigote viability [67]. Recent data from the kinome-wide deletion screen identify, together with the aforementioned kinases, a number of CRKs refractory to deletion (CRK2-*Lmx*M.05.0550, CRK9-*Lmx*M.27.1940 and CRK11-*Lmx*M.29.1780) and important CRK kinases for survival in the host [37]. Future enzymatic assays can be developed to further pharmacologically validate those results and to perform inhibitory screens for drug development. 

## 5. Glycogen Synthase Kinase 3 (GSK-3)

Glycogen synthase kinase 3 (GSK-3) is a multifunctional serine/threonine kinase found in all eukaryotes. It is a member of the CMGC family and closely related to CDKs [86]. In higher eukaryotes, GSK-3 is important in many cellular processes (Wnt signaling, cell cycle, differentiation, apoptosis, neuronal functions, etc.), while its deregulation has been linked to many human diseases such as cancer, diabetes and Alzheimer’s disease [86]. In trypanosomatids, two genes encode for two isoforms of GSK-3 (GSK-3s—short isoform and GSK-3l—long isoform). The short isoform in trypanosomatids has been validated pharmacologically or genetically as a potential drug target [26,27,87].

In *Leishmania*, GSK-3s has been validated by both pharmacological assessment [26,27] and genetic manipulation, where *Leishmania* promastigotes were refractory to GSK-3 deletion (Figure 2) [37], and these studies suggest that GSK-3s is an essential gene. Chemical inhibition of the kinase resulted in cell cycle deregulation, namely G1 phase arrest that was followed by an apoptosis-like death in the parasites (Figure 2) [26,27], suggesting that GSK-3s may have a direct or indirect role in cell cycle progression. Apart from the essentiality of the kinase, there are additional reasons that make this kinase a good drug target for antileishmanial drug design. Firstly, there are specific changes in the ATP binding pocket between the human and the parasite orthologues, i.e., the replacement of Gln185*_h_*_GSK-3__β_ to His155*_Ld_*_GSK-3s_ in the sugar-binding region, and the “gatekeeper” replacement of Leu132*_h_*_GSK-3__β_ to Met100*_Ld_*_GSK-3s_ [26] that can be exploited for selective inhibition. More specifically, the replacement of the leucine gatekeeper to a methionine which results in a larger entropic and desolvation cost upon inhibitor binding and the presence of the proton accepting His155*_L_*_GSK-3,_ could be exploited for designing bulkier inhibitors to enhance selectivity towards leishmanial over the human GSK-3 [26].

Secondly, the availability of the crystal structure of *L. major* GSK-3s (*Lmj*F18.0270) is anticipated to advance drug discovery efforts [33,88]. Moreover, there is a large availability of known *h*GSK-3 inhibitors that can be tested against the leishmanial GSK-3s. Many studies have focused on the repositioning of *h*GSK-3 inhibitors against the leishmanial homologue [26,27,70,72] (Table 1). In this context, the anti-parasitic action of an in-house indirubin library known to inhibit mammalian GSK-3 and CDKs was evaluated. Amongst the indirubins displaying potent antileishmanial activity, were those with 6-Br-substitutions on the indirubin backbone, a substitution known to greatly enhance the selectivity for mammalian GSK-3 over CDKs. Kinase assays revealed that the 5-Me-6-BIO was a potent inhibitor of *Ld*GSK-3s (*Ld*BPK_180270) (nM range), while it displayed potent anti-parasitic activity against *L. donovani* promastigotes and intracellular amastigotes (low micromolar range) [26]. In addition, 5-Me-6-BIO was several-fold more selective over the CRK3 homologue. Despite that selectivity, other 6-Br substituted analogues like 6-bromoindirubin-3′-oxime (6BIO), reversed selectivity from the respective mammalian kinases and targeted more potently CRK3 over GSK-3s. Thus, a new study was undertaken for understanding selectivity with new indirubin analogues and showed that the GSK-3s affinity of 6-BIO derivatives was enhanced upon 3′-substitution with bulky amino groups, namely piperazine or pyrrolidine [27]. This study confirmed that potent analogues (GSK-3s inhibitors, nM range) also possess important anti-parasitic activity against *L. donovani* promastigotes and intracellular *L. donovani* amastigotes at the low micromolar range [27].

Apart from indirubins, paullones which initially were characterised as CDK inhibitors were found to be excellent GSK-3 inhibitors [71]. For instance, Knockaert et al. showed that *in vitro* alsterpaullone inhibited the growth of *L. mexicana* promastigotes, while it prevented the parasite replication in macrophages [70]. The antileishmanial compounds maleimides [89,90] and lithium [91] were also found to inhibit the parasitic GSK-3s. In search for new scaffolds that inhibit GSK-3, another study that tested a group of compounds developed by GlaxoSmithKline known to possess antileishmanial action (*Leish*box), and an in-house library of human GSK-3β inhibitors, revealed that compounds belonging to the families thiadiazolidindione, halomethylketone, maleimide, benzoimidazole, *N*-phenylpyrimidine-2-amine and oxadiazole, inhibited *L*GSK-3s. With respect to *Leish*box, benzoimidazole, *N*-phenylpyrimidine-2-amine and oxadiazole were uncovered as new scaffolds for *Ld*GSK-3s (Table 1) [72].

Although most of these compounds (Table 1) are also competitive inhibitors of the mammalian GSK-3, the selectivity index between *Leishmania* and human cell cytotoxicity appears in most cases to be high due to the tolerance of the host kinase inhibition in adult mammals [92,93]. This redundancy of the mammalian homologue kinase as well as the aforementioned arguments, highlights the kinase as an excellent candidate for targeted drug discovery.

## 6. Dual-Specificity Tyrosine-Regulated Kinases (DYRKs)

The dual-specificity tyrosine-regulated kinases, namely DYRKs, belong to the CMGC group of kinases [94] and as their name reveals, they possess dual specificity. More specifically, DYRKs demonstrate an autophosphorylation activity of a tyrosine residue in their activation loop, and a serine/threonine kinase activity towards their substrates [95]. DYRKs are expressed in all eukaryotes [96]. YAKs and minibrain kinases are DYRK family members expressed in lower eukaryotes (yeast) and nematodes, respectively [95,97,98,99]. In higher eukaryotes, DYRK family members are categorized into three subfamilies based on their kinase domain homology: the HIPKs (homeodomain interacting protein kinase) that are regulators of a variety of stress signals [100], the pre-mRNA processing protein 4 kinases (PRP4s) that are involved in transcriptional regulation [101] and the DYRKs [96]. DYRKs are further divided to class I (DYRK1A and DYRK1B) and class II (DYRK2, DYRK3 and DYRK4) [96]. DYRK subfamily members are multifunctional kinases involved in a variety of cellular processes in mammalian cells, such as stasis regulation, differentiation, proteosomal degradation, chromatic remodeling, gene expression and others [96]. The most studied members in mammalian cells are the two closely related and multifaceted DYRK1 kinases (DYRK1A and DYRK1B). DYRK1A is implicated in Down syndrome pathology and neurodegenerative disorders, whereas DYRK1B deregulation is associated with metabolic syndrome and cancer [102,103,104,105,106,107].

*Leishmania* encodes for 8 DYRK family members [108]. Five belong to the aforementioned subfamilies [*L. infantum* orthologues DYRK subfamily (DYRK1-*Lin*J.15.0180, *Lin*J.14.0890, DYRK2-*Lin*J.33.1890) HIPKs (*Lin*J.19.0360), Prp4s (*Lin*J.36.440)], while 3 members cluster away from these groups (PK4-*Lin*J.21.210, *Lin*J.14.1140, *Lin*J.35.180). These results suggest that a new categorization of the DYRK family is needed and requires the inclusion of protozoan genes [108]. Interestingly, class I DYRKs in *Leishmania* are more closely related to mammalian DYRKs than to lower eukaryote YAKs [108].

Recently, attempts were made by Loaec et al. to investigate DYRK inhibition by 10 marine natural products which shared a 2-aminoimidazolone scaffold against mammalian and leishmanial DYRKs [77]. In the latter study, the authors cloned and expressed three *L. donovani* DYRK kinases (*LD*BPK_351850, *LD*BPK_212010-PK4 and *Ld*DYRK1) and one *L. major* kinase (*Lm*DYRK2—*LMJ*F_33_1830). Leucettamine B, its derivative Leucettine L41 and Polyandrocarpamine A, displayed medium or high activity against *Lm*DYRK2 (IC_50_: 4.2, 2.9 and 5.9 μM, respectively) and/or *Ld*DYRK1 (IC_50_: 6.9, 0.82 and 1.1 μM, respectively), while the compounds Hymenialdisine and Spongiacidin B seem to inhibit effectively *Lm*DYRK2 (IC_50_: 0.38 and 9 μM, respectively), *Ld*DYRK1 (IC_50_: 0.042 and 0.5 μM, respectively) and *LD*BPK_351850 (IC_50_: 0.021 and 0.3 μM, respectively) (Table 1, Figure 2) [77]. However, the anti-parasitic activity of *Leishmania* DYRK inhibitors against *Leishmania* promastigotes and intracellular amastigotes has not been evaluated yet and merits further investigation.

Moreover, the role of *L. infantum* DYRK1 (*Lin*J.15.0180) has been recently investigated [108]. The deletion of DYRK1 by gene replacement rendered viable *L. infantum* promastigotes, showing that parasites can compensate for its loss [108]. Furthermore, *Lin*DYRK1 deletion mutants displayed defects in the stationary phase and in metacyclogenesis, followed by significant reduction in survival within peritoneal macrophages (Figure 2) [108]. Another study, however, in *L. mexicana* using CRISPR-CAS9 technology failed to isolate a gene deletion mutant for DYRK1 (*Lmx*M.15.0180) [37]. This could be due to differences between species or compensatory mutations or because of the background expression levels of other DYRK kinases that may be able to compensate for the loss of DYRK1. In addition, it was shown that *Lin*DYRK1 over-expressing parasites displayed a decrease in proliferation and in cell cycle re-entry, suggesting a role as a stasis regulator similar to its mammalian homologues. *Lin*DYRK1 has a distinct localization in the cytoplasm, flagellar pocket area and the endolysosome [108]. The latter localization suggests that DYRK1 is a multifaceted kinase, while its presence in the endosomal compartment is reminiscent of a known role for mammalian DYRK1A in endocytosis and vesicle recycling [109,110]. A similar localization to DYRK1 was also shown in *L. mexicana* for another DYRK family member, namely *LmxM*.14.0 70 (*LinJ*.14.1140 orthologue of *L. infantum*), suggesting that the two kinases could participate in the same cellular processes. In addition, the most closely related homologue of DYRK1, *Lmx*M.14.830 was refractory to deletion, suggesting that this kinase could be essential for viability and thus could be a potential drug target. Another important DYRK-like kinase, namely PK4 (*Lmx*M.21.1650), was shown to be required for differentiation and/or survival in the mammalian host only [37]. Overall, *Leishmania* DYRKs are interesting kinases as potential drug targets, and the study of these kinases will provide important information on to the life-cycle of the parasite. The above data together suggest that DYRK1 and other family members like *Lmx*M.14.830 could serve as potential drug target candidates.

## 7. Mitogen-activated Protein Kinases

Mitogen-activated protein kinases (MAPKs) are members of the CMGC of serine/threonine kinases [38] found in all eukaryotes, and are known to mediate signal transduction from external stimuli, such as mitogens, pro-inflammatory cytokines, heat shock and other stress stimuli [111]. The mammalian MPK family of kinases includes three subfamilies: the extracellular signal-regulated kinases (ERKs), the c-Jun N-terminal kinases (JNKs) and the p38 mitogen-activated protein kinases (p38s). ERKs are known to be activated by growth factors and mitogens, whereas cellular stresses and inflammatory cytokines activate JNKs and p38s [112]. Classical MAPKs become activated by phosphorylation in their activation loops, which contain a characteristic TxY motif [113]. This phosphorylation is normally part of a signaling cascade with upstream kinases belonging to the STE family (“sterile”), referring from null mutants that resulting in sterile yeast. This cascade starts by the stimulation of MAP kinase kinase kinases (M3Ks), that phosphorylate and activate MAP kinase kinases (M2Ks), which finally phosphorylate and activate MAP kinases (MAPKs), for the regulation of various cellular activities like cell proliferation, differentiation, stress response, infectivity and apoptosis [111,114].

*Leishmania* parasites encode for 17 mitogen-activated protein kinases (MAPKs), 5 putative MAP2Ks (STE) and around 25 MAP3Ks (STE) [35,115]. Of all leishmanial protein kinase families, MAPKs are the most commonly studied, and their known role in coupling extracellular signals with cell survival has put them in the spotlight as an important source of potential drug targets. In this context, some members of MAPK family have already been validated as potential targets. Amongst these, is *Lmx*MPK1 (*Lmx*M.20_36.6470), a kinase required for intracellular parasite survival [116]. *Ld*MPK1 phosphorylates HSP90 and HSP70, thus may regulate the stability and activity of the foldosome [117,118] specific to the *Leishmania* infectious stage [119]. Thus, deregulation and inhibition of its activity may result in increased thermo-sensitivity and decreased intracellular parasite viability (Figure 2). It also appears that MPK1 negatively regulates the expression of P-glycoprotein-type efflux pumps in *L. donovani* promastigotes [120], thus this kinase activity maybe associated with drug resistance. In addition to *Lmx*MPK1, *Lmj*MPK2 (*Lmj*F.36.0720), which is required to establish infection in mice, seems to be essential in amastigotes and could serve as a potential drug target [121]. *Lmj*MPK2 also controls osmoregulation and Sb(III) uptake by phosphorylating and stabilizing aquaporin [121]. Thus, the activity of *Leishmania* MPK2, like MPK1, is inversely associated with the acquisition of drug resistance [121]. Confirmation about the importance of *Lmx*MPK1 and *Lmx*MPK2 in intracellular survival, came by the study of Baker et al., who showed that these kinases are important for differentiation in both insect vector and mammalian host [37]. Moreover, it was shown that an arginine deprivation response induced during macrophage infection is mediated through an MPK2-dependent signaling cascade, thus providing evidence for its mode of action in intracellular parasites [122].

In addition to *Leishmania* MPK1 and MPK2, reverse genetics approaches revealed a role for *Lmx*MPK3 (*Lmx*M.10.0490) and *Lmx*MPK9 (*Lmx*M.19.0180) in the promastigote flagellum length regulation [123,124]. *Lmx*MPK9 deletion mutants had a long flagellum whereas *Lmx*MPK9 over-expressing parasites had a shorter paraflagellar rod protein PFRA [124]. Moreover, it was shown that *Lmx*MPK9 is important for the parasites’ colonization in the sandfly [37]. On the other hand, *Lmx*MPK3 deletion mutants had a shorter flagellum (Figure 2) [123], a similar phenotype to null mutants of *Lmx*MPKK1, the activating kinase of *Lmx*MPK3 [125]. Although *Lmx*MPK3 is not essential for parasite viability, small molecule inhibitors have been identified, as this kinase is important for *Leishmania* transmission [80]. Shweta Raj et al. showed that the plant-based natural compounds genistein and chrysin (Table 1) inhibited the kinase, and at the same time, minimized the parasitic load of infected macrophages (Figure 2) [80]. However, genistein and chrysin may act on enzymes of the macrophage or other parasitic enzymes, and hence further work is necessary to ascertain that the reduction of infectivity is due to the inhibitory effect of *Ld*MPK3. Furthermore, as *L. donovani* MPK3 (*Ld*BPK_100540) crystal structure is available from the Protein Data Bank (PDB), drug design against MPK3 is more feasible [126,127].

Genetic analyses of *L. major* MAPKs (termed *Lma*MPKs) revealed amastigote-specific phosphorylation and activity of *Lma*MPK4, *Lma*MPK7 and *Lma*MPK10 [128] and established a role of *Lma*MPK7 in translational control of amastigote proliferation [129]. Wang et al. generated *Lmx*MPK4 null mutants only in the presence of an extrachromosomal copy [130], while Dacher et al. performed a facilitated approach of MPK4 deletion mutants in the presence of a plasmid susceptible for negative selection that expresses MPK4, which confirmed that this kinase is essential for viability [131] and hence validated MPK4 as a potential drug target. In addition, a mutant expressing MPK4 with an altered ATP binding pocket (K59R) showed defects in metacyclogenesis (Figure 2), linking MPK4 activity with parasite differentiation [131]. Located primarily in the lysosome [37], *Lmx*MPK4 is activated upon phosphorylation by the STE7 *Lmx*MKK5. The finding of *Lmx*MPK4′s *in vitro* activator formed the basis on which screens for identifying small molecule *Lmx*MPK4 inhibitors can be established [132]. Despite the knowledge of the activator kinase, only *in silico* screening has been performed. In this context, Shweta Raj et al. performed an *in silico* study to identify potent *Ld*MPK4 inhibitors among 110 natural inhibitors of *Leishmania* parasite and demonstrated that genistein and chrysin are potential lead molecules for targeting the kinase [133]. Moreover, Saravanan et al. virtually screened 2654 compounds from an NCI Diversity set against the human ERK2 and the *Lmx*MPK4 and identified potential inhibitors with higher affinity against *Lmx*MPK4 [134]. The authors also demonstrated that the *Lmx*MPK4 ATP binding domain, although highly conserved, possesses minor but potentially important structural differences to the homologous human ERK2. More specifically, ligands bind to the *Lmx*MPK4 hydrophobic inner part of the cavity to the residues Val26, Leu91, Met93, and Cys155 and form, at the same time, hydrogen bonds with anchoring key residues such as Tyr96 which surround the ATP binding cavity [134]. Taking into consideration the above characteristics, *Lmx*MPK4 is an important candidate for designing new scaffolds of targeted drugs with high affinity against the specific kinase.

*Lmx*MPK5 (*Lmx*M.29.2910) and *Lmx*MPK10 (*Lmx*M.10.0200) also seem to play a crucial role in the amastigote form of the parasite and thus could serve as potential drug targets in order to stop the parasites from hijacking the macrophages and infecting the host. Mpk5 gene was successfully deleted in *L. mexicana* and the generated null mutants although persisted in the inoculation site upon infection of BALB/c mice, they displayed decreased ability to cause lesions (Figure 2) [135]. Moreover, *Lmx*MPK10 has been shown to be implicated in amastigote differentiation, while it also displays stage-specific activity important in promastigote and amastigote differentiation (Figure 2) [136]. Interestingly, it was also shown that *Lma*MPK10 possesses a potential C-terminal autoinhibitory mechanism which could be exploited for the discovery of molecules interfering with MPK10 [81]. This autoinhibitory mechanism seems crucial for the stage-regulated activity and parasite viability [136]. The stage-regulated activity of *Leishmania* MPK10 was confirmed for the *L. mexicana* orthologue [37]. A crystal structure of *Lma*MPK10 is available, highlighting the significant differences compared to human p38 kinase in the catalytic pocket, as well as the potentially regulatory sites in the N-terminal lobe [81].

Moreover, Baker et al. showed that MPK15 (*Lmx*M.32.2070) is likely important in the differentiation and/or survival in the mammalian host [37]. The same study also revealed that *L. mexicana* parasites were refractory for the deletion of 8 STE genes, including MPKK4 and MPKK5 [37]. In summary, the above results show that members of these groups of kinases (MPKs and STEs) merit in-depth investigation for drug discovery efforts and for their role in differentiation and environmental sensing.

## 8. Casein Kinases

The term “casein kinase” (CK) applies to three unrelated categories of protein kinases, denoted by the acronyms CK1, CK2 (casein kinase 1 and 2) and G-CK (Golgi–Casein kinase or Fam20C), sharing the ability to readily phosphorylate casein *in vitro* [137]. CK1 is a family of monomeric, Ser/Thr protein kinases ubiquitously expressed and present in all eukaryotes. Seven isoforms of human CK1 exist (α, β, γ1, γ2, γ3, δ and ε), displaying highly conserved kinase domains [137]. CK1s are involved in various cellular processes, such as in Wnt and Hedgehog signalling, circadian rhythm, nucleo-cytoplasmic shuttling, DNA repair, DNA transcription and others [138,139,140]. CK1s are known to have a wide range of substrates [138], while they are involved in hierarchical phosphorylation events, acting either as a priming or as a processive kinase [141,142]. Thus, deregulation of CK1 isoforms is implicated in several diseases, such as neurodegenerative, sleeping disorders, and neoplasia [137]. CK2 is one of the most pleiotropic protein kinases, with dual specificity, which phosphorylates either serine/threonine or tyrosine residues and is composed of two catalytic (CKA1, CKA2) and two regulatory subunits (CKB1 and CKB2), respectively. CK2, like CK1, is responsible for the generation of a large proportion of the phosphoproteome, phosphorylating plethora of substrates. CK2 is involved in many processes like cell proliferation and survival, apoptosis, angiogenesis and other activities [143]. The third member of the CKs, FAM20C is an atypical kinase that also generates a high number of the phosphoproteome and a pleotropic enzyme [137]. As human casein kinases are implicated in many processes, they have become the focus of drug discovery efforts, usually against cancer and neurodegenerative diseases [137].

**Figure 3:**
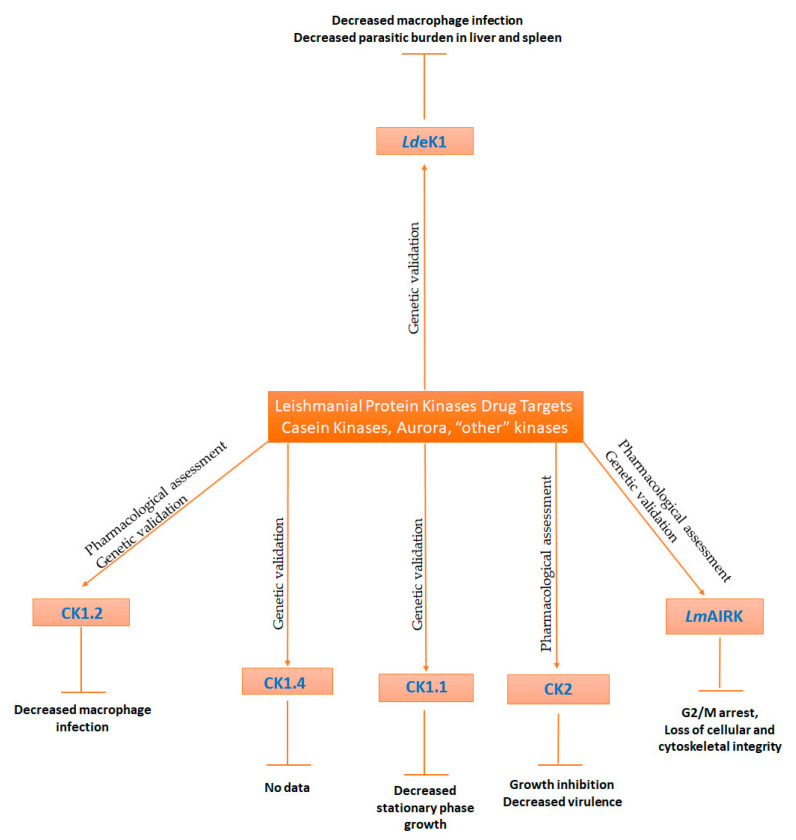
Leishmanial Protein kinases from the Casein kinase family, Aurora and “other” kinase families, that could serve as drug targets. The ePKs displayed have been genetically and/or pharmacologically validated. The effects on their biological role and/or on their virulence upon pharmacological and/or genetic inhibition is also displayed in the diagram.

Out of the three families of casein kinases, only CK1 and CK2 are found in *Leishmania*, while FAM20C is not present. *Leishmania* parasites have an expanded CK1 family relative to their kinome, with 6 members (CK1.1-*Lmx*M.34.1000, CK1.2-*Lmx*M.34.1010, CK1.3-*Lmx*M.04.1210, CK1.4-*Lmx*M.27.1780, *Lmx*M.25.1580 and *Lmx*M.29.3470 in *L. mexicana*) [35,37,73]. Sacerdoti-Sierra and Jaffe showed that released ectoproteins from *L. major* promastigotes displayed CK1 activity [144] and later on the finding were confirmed as at least one member of the family, namely CK1.2, was found in the secretome [145,146]. It has been demonstrated that the *Leishmania* CK1 phosphorylated human IFNAR1, a receptor implicated in interferon signalling and hence it was suggested that it may be implicated in macrophage function and immune response [147]. Furthermore, Rachidi et *al.* with the use of a specific CK1 inhibitor D4476 (4-[4-(2,3-dihydro-1,4-benzodioxin-6-yl)-5-(2-pyridinyl)-1*H*-imidazol-2-yl]benzamide) (Table 1), pharmacologically validated CK1.2 as a potential drug, as the inhibitor blocked promastigote growth, compromised axenic amastigote viability and decreased the number of intracellular *L. donovani* and *L. amazonensis* amastigotes in infected macrophages (Figure 3) [73]. In addition, the study of Baker et al. showed that *L. mexicana* promastigotes were refractory to CK1.2 deletion, suggesting that CK1.2 maybe essential for viability [37]. It was also shown that *Leishmania* CK1 has the ability to phosphorylate *Leshmania* HSP83, an important protein for stage regulation and *Leishmania* viability [118]. The fact that *Leishmania* CK1.2 is important for viability highlights this kinase as an important drug target candidate to treat leishmaniasis, and several CK1.2 inhibitors have been identified (Table 1). Apart from D4476, inhibition of the kinase by trisubstituted pyrrole, blocked promastigote growth [148]. Durieu et al. screened 5018 compounds to assess their antileishmanial activity against promastigotes and intracellular amastigotes, as well as their activity against CK1.2 [74]. They identified two compounds, PP2 (1-tert-butyl-3-(4-chlorophenyl)-1 h-pyrazolo[3,4-d]pyrimidin-4-amine) and an indirubin analogue (compound 42), that targeted the kinase and displayed potent antileishmanial activity with a high selectivity index (>10) against murine macrophages (Table 1) [74]. Thus, targeted inhibition of the parasites’ essential kinase CK1.2 could have dual activity for treating leishmaniasis, as it would lead to parasite death and it would cease the establishment of the infection in the mammalian host.

In addition to CK1.2, *Lmx*CK1.4 isoform was also shown to be essential for parasitic viability, as no CRISPR-cas9 mutants could be generated (Figure 3) [37]. CK1.4 is the only CK1 isoform that is unique to *Leishmania* [35,37,149] and therefore it is considered a potential chemotherapeutic target. Although CK1.4 is localized in the flagellar pocket of *L. mexicana* promastigotes, it is also secreted like CK1.2 and is considered a potential virulence factor [37,73,149]. This theory is verified by the fact that overexpression of the kinase in *L. donovani* promastigotes resulted in significantly higher infections of mouse peritoneal macrophages compared to wild-type parasites [149]. Subsequently, CK1.4 isoform has to be further investigated, as it plays a crucial role in both the parasitic survival and the ability to infect the host macrophages [149].

Another *Leishmania* isoform, CK1.1 was not found to be essential for *Leishmania* survival or axenic amastigote differentiation. It was demonstrated that CK1.1 was a low-abundance protein present in promastigotes and in amastigotes. In the same study, however, there was a link between CK1.1 and stationary phase, as CK1.1 null parasites reached lower cell density (Figure 3) [150].

Along with CK1 kinases, *Leishmania* parasites encode for two CK2 isoforms. Many studies have described CK2 activity in *Leishmania* species, such as *L. tropica* [76], *L. major* [144], *L. amazonensis* [151] and *L. donovani* [152]. CK2 isoforms have been reported to be extensively distributed in *Leishmania* from the surface of the parasitic cells to the cytoplasm, the nucleus and as a secreted kinase, possessing ecto-CK2, intracellular CK2 and secreted CK2 activity, respectively [37,75,76,151,152]. It actually seems that CK2 is located in different cell compartments and it responds to different extracellular protein targets, suggesting that CK2, although proven dispensable for the parasite’s viability, can serve as a chemotherapeutic target due to its multifunctionality. It was shown that CK2 is vital for the growth of the virulent parasites, as CK2 inhibition with heparin, DRB (5,6-dichlorobenzimidazone-1-*β*-D-ribofuranoside) and TBB (4,5,6,7-tetrabromobenzotriazole) resulted in growth inhibition only in a virulent parasite strain (Table 1, Figure 3) [75]. Moreover, in *L. major* and *L. amazonensis*, the kinase seems to influence not only the growth and morphology of the parasites, but also the infection and/or survival within macrophages *in vitro* and *in vivo*, in a BALB/c animal model (Figure 3) [76,151]. It has been observed that secreted CK2 seems to play an important role during the host infection. In *L. chagasi* (or *L. infantum)*, mRNA levels of the kinase differ between the different life stages of the parasite, a fact that also indicates its importance of the parasite survival in the host. There is also an indication that *Lc*CK2A controls the gene expression during the parasite’s life cycle. Highly virulent *L. braziliensis* promastigotes secrete increased levels of CK2 whose substrates are presented by macrophages or human serum, while iNOS and arginase, two mammalian enzymes responsible for the parasite survival in the macrophages, seem to activate *L*CK2 [144,152]. Dutra et al. demonstrated that platelet activating factor (PAF) also activates the secreted and the membrane-bound CK2 [76] and induces parasite differentiation in *L. tropica* [153]. All the above advocate that CK2 activation promotes parasite–host interactions. Moreover, CK2 inhibitors TBB and DRB inhibited the stimulation of macrophage infection, suggesting that CK2 can also serve as a target in order to cease parasite invasion and differentiation in the macrophages [76].

## 9. Aurora Kinases

Aurora kinases are known mediators of the cell division cycle in eukaryotes [154]. Since they are considered important mitotic serine/threonine kinases, Aurora kinases are well studied in a variety of organisms. In *L. mexicana*, three Aurora kinases have been identified (AUK3-*Lmx*M.26.2440, AUK1/AIRK-*Lmx*M.28.0520, AUK2-*Lmx*M.08_29.1330) [37], while in *L. major*, the role of one Aurora kinase member (*Lm*AIRK-AUK1 homologue) has been investigated [155,156,157]. A computational analysis identified the putative Aurora-like kinase in *L. donovani* (*Ld*AIRK), the *Lm*AIRK homologue, as a potential drug target [78]. Knockout *Ld*AIRK parasites could not be generated, a fact that strengthens the hypothesis that the kinase is important for viability [78]. Another important feature of the kinase is that although in mammalian cells, Aurora kinases play a role in mitosis, cytokinesis and chromosome segregation, in *L. major*, one gene of Aurora kinase, namely *Lm*AIRK, may regulate all these processes [156]. With a main nuclear localization throughout the cell-cycle, *Ld*AIRK seems to temporarily migrate from the cytoplasm to the nuclear periphery and ultimately to the spindle poles during the early mitosis and post mitosis [78]. Based on the importance of the role that the kinase plays in the life cycle of the parasite, it emerged as a potential chemotherapeutic target. The advantage of repositioning mammalian Aurora kinase inhibitors in order to evaluate their antileishmanial activity and to assess their inhibitory activity against the leishmanial homologue, provides an advantage for discovering new treatment that targets the Aurora kinase in *Leishmania*. Among the repositioned Aurora inhibitors, hesperadin (Table 1) was found to possess a strong antileishmanial activity, as parasites incubating with the inhibitor displayed an accumulation of cells in G2/M phase that finally led to the loss of cellular and cytoskeletal integrity (Figure 3). The above results imply that *Ld*AIRK has a crucial role in cytokinesis and cell-cycle progression [78,79].

## 10. Other Kinases

Apart from the parasite kinases mentioned above, an increasing number of studies are shedding light on the role of new kinases outside the aforementioned groups. For instance, a new gene was recently identified in *L. donovani*, namely *Ld*-RAC/Akt-like gene (and its *L. panamensis* orthologue *Lp*-RAC/Akt-like gene), which expresses a protein with major mammalian Akt hallmarks, including the typical pleckstrin homology domain, protein kinase and AGC kinase domains, but with only 26.5% identity with mammalian Akt1 [158,159]. This kinase is considered a putative molecular target to treat leishmaniasis, and there is evidence that is essential for parasitic survival [82]. Thus far, Devki Nandan et al. showed that Miransertib, a mammalian Akt inhibitor, possesses inhibitory activity against both *Leishmania* promastigotes and intracellular amastigotes and indicated that the homologue leishmanial Akt is the probable target of the inhibitor (Table 1) [82].

Moreover, the *L. donovani* eukaryotic initiation factor 2 alpha (eIF2α) kinase (*Ld*eK1), which regulates translation under stress conditions (nutrient starvation, acidic pH, high temperature) through the phosphorylation of eIF2α, has emerged as a candidate for vaccine development against leishmaniasis [160,161]. The above hypothesis was supported by the fact that *Ld*eK1 modulates the immune response towards host protection as *in vitro* and *in vivo* infection, with *Ld*eK1 negative mutants resulting in reduced parasite burden in macrophages and in splenic/hepatic load, respectively (Figure 3), and in enhanced pro-inflammatory cytokines and nitric oxide levels with parallel reduced levels of TH_2_ and Treg populations [160].

The Ser/Thr kinase *Lmj*F.22.0810 has been characterized as a likely essential protein for viability and seems to be involved in the drug response of *Leishmania.* More specifically, its sensitivity towards aminoglycosides such as paromomycin (Table 1) is potentially correlated to the mechanism of drug resistance in *Leishmania* parasites [83]. Another kinase (*Ld*_f1), a homologue of bacterial-zeta-toxin was recently identified in *L. donovani* possessing UNAG kinase and ATP-binding activity. *Ld*_f1 is tightly correlated with cell death and is a putative target candidate for development novel antileishmanial chemotherapeutics [162].

## 11. Future Directions—Concluding Remarks

On the basis of the drawbacks of the current chemotherapy for the treatment of leishmaniasis (emerging resistance, cost, toxicity), the discovery of new antileishmanial drugs and the development of new treatments may be urgent, but appears challenging. Protein kinases serve as ideal targets for rational drug design. In general, eukaryotic protein kinases play a fundamental role in the survival and/or virulence of *Leishmania* parasites, and this fact places them in the center of attention for designing novel antileishmanial small molecule inhibitors. The understanding of the role of each specific parasitic kinase can expand our knowledge on the mechanisms used by *Leishmania* parasites to adapt in the host and to establish infection, and can be used as an exploitable tool to fight the disease. Although there are new studies that focus on leishmanial ePKs and in their potential role as molecular targets for rational drug design, more efforts are needed in the field. The availability of the crystal structures of certain leishmanial kinases could speed up the discovery of molecules inhibiting their activity, with relevance to antileishmanial drug development. Moreover, more efforts are needed to establish screening platforms of important kinases, and technical issues need to be addressed, including the knowledge of substrates, the development of purification protocols enabling the isolation of active form of the protein. All the above knowledge will aid towards the discovery of novel and specific antileishmanials, and in combination with new technologies such as nanotechnology approaches for optimal drug delivery, it is expected to provide us with a new perspective in the battle against leishmaniasis.

## Figures and Tables

**Table 1:** Identified inhibitors targeting leishmanial eukaryotic Protein Kinases.

Target (Protein Kinase)	Molecules/Drugs	Anti-Parasitic Activity	References
**CRK3**	2,6,9-Trisubstituted purines (including C-2-alkynylated purines)	YES	Řezníčková et al. [61] Grant et al. [25] Grant et al. [68]
	Triazolopyridine inhibitors	NO	Cleghorn et al. [62]
	2,6-disubstituted purines and corresponding 3,7-disubstituted pyrazolo (4,3-)pyrimidines	YES	Jorda et al. [65]
	Azapurine compounds	NO	Walker et al. [63]
	Thiazole compounds	Moderate	
	Indirubin analogues (i.e., 6-BIO and indirubin-3′-monoxime)	YES	Xingi et al. [26] Hoessel et al. [69]
	Paullones	toxic to MF	Grant et al. [25]
	Staurosporine derivatives	toxic to MF	
**CRK12**	Pyrazolopyrimdines	YES	Wyllie et al. [66]
**GSK-3**	Indirubin analogues (mostly 3′bulky-6-BIO analogues)	YES	Xingi et al. [26] Efstathiou et al. [27]
	Paullones (alsteroaullone)	YES	Knockaert et al. [70,71]
	Thiadiazolidindione	YES	Martinez de Iturrate et al. [72]
	Halomethylketone	YES	
	Maleimide	YES	
	Benzoimidazole	YES	
	N-phenylpyrimidine-2-amine	YES	
	Oxadiazole	YES	
**CK1.2**	4-[4-(2,3-dihydro-1,4-benzodioxin-6-yl)-5-(2-pyridinyl)-1H-imidazol-2-yl]benzamide (D4476)	YES	Rachidi et al. [73]
	PP2 (1-tert-butyl-3-(4-chlorophenyl)-1 h-pyrazolo [3,4-d]pyrimidin-4-amine) and an indirubin analogue (compound 42)	YES	Durieu et al. [74]
**CK2**	TBB (4,5,6,7-tetrabromobenzotriazole)	YES	Zylbersztejn et al. [75] Dutra et al. [76]
	DRB (5,6-dichlorobenzimidazone-1-*β*-d-ribofuranoside)	YES	
	Heparin	YES	Zylbersztejn et al. [75]
***Ld*DYRK1 and/or *Lmx*DYRK2**	2-aminoimidazolone scaffold (Leucettamine B, its derivative Leucettine L41, Polyandrocarpamine A, Hymenialdisine and Spongiacidin B)	No data	Loaec et al. [77]
***Ld*AIRK**	Hesperadin and Hesperadin analogs	YES	Chhajer et al. [78] Patel et al. [79]
**MPK3**	Genistein, Chrysin	YES	Raj et al. [80]
**MPK10**	SB203580	No data	Horjales et al. [81]
***Ld*-RAC/Akt-like**	Miransertib	YES	Nandan et al. [82]
***Lmj*F.22.0810**	Aminoglycosides (paromomycin)	YES	Vacas et al. [83]
***Lmx*PYK**	Suramin	YES	Morgan et al. [84]
	Saccharin derivatives	No data	Morgan et al. [85]
